# Admission serum potassium concentration and long-term mortality in patients with acute myocardial infarction: results from the MONICA/KORA myocardial infarction registry

**DOI:** 10.1186/s12872-017-0635-x

**Published:** 2017-07-24

**Authors:** Miriam Giovanna Colombo, Inge Kirchberger, Ute Amann, Margit Heier, Christian Thilo, Bernhard Kuch, Annette Peters, Christa Meisinger

**Affiliations:** 1 MONICA/KORA Myocardial Infarction Registry, Central Hospital of Augsburg, Augsburg, Germany; 2 Institute of Epidemiology II, Helmholtz Zentrum München, German Research Center for Environmental Health (GmbH), Neuherberg, Germany; 3 Department of Internal Medicine I – Cardiology, Central Hospital of Augsburg, Augsburg, Germany; 4 Department of Internal Medicine/Cardiology, Hospital of Nördlingen, Nördlingen, Germany

**Keywords:** Myocardial Infarction, Potassium, Hypokalemia, Hyperkalemia, Mortality

## Abstract

**Background:**

Conflicting with clinical practice guidelines, recent studies demonstrated that serum potassium concentrations (SPC) of ≥4.5 mEq/l were associated with increased mortality in patients with acute myocardial infarction (AMI). This study examined the association between SPC and long-term mortality following AMI in patients recruited from a population-based registry.

**Methods:**

Included in the study were 3347 patients with AMI aged 28–74 years consecutively hospitalized between 1 January 2000 and 31 December 2008 and followed up until 31 December 2011. Patients were categorized into five SPC groups (<3.5, 3.5 to <4.0, 4.0 to <4.5, 4.5 to <5.0, and ≥5.0 mEq/l). The outcome of the study was all-cause mortality. Cox regression models adjusted for risk factors, co-morbidities and in-hospital treatment were constructed.

**Results:**

In our study population, 249 patients (7.4%) had a low SPC (<3.5 mEq/l) and 134 (4.0%) patients had a high SPC (≥5.0 mEq/l). Patients with SPC of ≥5.0 mEq/l had the highest long-term mortality (29.9%) and in the adjusted model, their risk of dying was significantly increased (HR 1.46, 95% CI 1.03 to 2.07) compared to patients with SPC between 4.0 and <4.5 mEq/l. Analyses of increasing observation periods showed a trend towards a higher risk of dying in patients with SPC between 4.5 and <5.0 mEq/l.

**Conclusion:**

An admission SPC of ≥5.0 mEq/l might be associated with an increased mortality risk in patients with AMI. Patients with an admission SPC between 4.5 and <5.0 mEq/l might have an increased mortality risk in the first few years following AMI.

## Background

Hypo- and hyperkalemia have been shown to increase cardiovascular and total mortality in patients with acute myocardial infarction (AMI) [1–3]. Hypokalemia refers to a serum potassium concentration (SPC) of <3.5 mEq/l, occurs frequently in hospitalized patients [1] and is associated with ventricular arrhythmias as well as an overall poor prognosis after cardiovascular events [2, 4]. Hyperkalemia is defined as a SPC of >5.0 mEq/l and can have a variety of adverse consequences, such as cardiac arrhythmias, in patients hospitalized after a cardiovascular event [3]. In patients with AMI recommended SPC are between 4.0 and 5.0 mEq/l [5, 6] or above 4.5 mEq/l [7].

In contrast to clinical practice guidelines [4–7], recent studies in patients with AMI concluded that a SPC of ≥4.5 mEq/l was associated with an increased in-hospital and 3-year mortality, respectively [8–10]. To examine whether these findings are valid for a longer observation period, we analyzed the association between SPC and long-term mortality in patients recruited from a population-based myocardial infarction registry.

## Methods

### Data source and study population

As part of the World Health Organization (WHO) project MONICA (Monitoring Trends and Determinants in Cardiovascular disease) the population-based Augsburg Myocardial Infarction Registry was established in 1984 [11]. MONICA was terminated in 1995 and the registry became part of the KORA (Cooperative Health Research in the Region of Augsburg) framework. Since the registry commenced, all cases of coronary death and non-fatal AMI of the 25- to 74-year old study population in the city of Augsburg and two adjacent counties (about 600,000 inhabitants) have been continuously registered. Patients admitted to one of the eight hospitals in the study area were included. Methods of case identification, diagnostic classification of events as well as data quality control have been described in detail elsewhere [11, 12]. Data collection and follow-up questionnaires have been approved by the ethics committee of the Bavarian Medical Association (Bayerische Landesärztekammer) and have been performed in accordance with the Declaration of Helsinki. All study participants gave written informed consent.

Included in our cohort study were all patients with a first ever AMI consecutively registered between 1 January 2000 and 31 December 2008, whose survival time exceeded 28 days after AMI. Patients were followed up until December 2011. From 4429 patients, we excluded those with missing SPC (*n* = 164) as well as those with incomplete data on any of the covariates included in our Cox regression models (*n* = 918). The final study population comprised 3347 male and female patients aged 28–74 years with a first ever AMI, who survived more than 28 days after the event.

### Data collection

Interviews with study participants were conducted by trained study nurses during hospital stay using a standardized questionnaire [13, 14]. Patient demographics, risk factors and co-morbidities were covered during the interviews. Laboratory values, AMI characteristics, medical and drug treatment as well as in-hospital complications were obtained from the patients’ medical record.

Patients’ SPC was determined at hospital admission and expressed in mEq/l. Patients were divided into five groups according to their SPC: < 3.5, 3.5 to <4.0, 4.0 to <4.5, 4.5 to <5.0, ≥5.0 mEq/l.

Renal function was assessed by calculating the estimated glomerular filtration rate (eGFR) using the Modification of Diet in Renal Disease (MDRD) study equation (eGFR (ml/min/1.73 m^2^) = 186.3 × (serum creatinine^−1.154^) x (age^−0.203^) × 0.742 (if female) × 1.212 (if black)) [15]. Since creatinine values were only available from 2005 onwards, missing values were incorporated in the analyses as part of a dummy-coded variable. An eGFR of <60 ml/min/1.73m^2^ indicates renal impairment and is independently associated with increased all-cause as well as cardio-vascular mortality [16, 17]. Therefore, eGFR values were classified into three categories: <60, ≥60 ml/min/1.73m^2^, and missing values.

Whether patients had been previously diagnosed with angina pectoris, hypertension, hyperlipidemia, diabetes and stroke (yes/no) as well as patients’ smoking status (smoker/ex-smoker/never-smoker) was determined during the interviews and, with the exception of stroke and smoking status, confirmed by chart review. Hemoglobin and glucose concentration were measured at hospital admission. The highest value of creatine kinase-myocardial band (CK-MB) measured during hospital stay was included in our analyses to serve as a marker for the extent of myocardial injury [18]. Whether any in-hospital revascularization (coronary artery bypass surgery, percutaneous coronary intervention (PCI) or thrombolysis) was performed during hospital stay was included in the analysis as a single covariate (yes/no). Furthermore, treatment with the following medications at hospital discharge were documented (yes/no): antiplatelet agents, beta-blockers, angiotensin-converting enzyme inhibitors (ACEIs) or angiotensin-receptor blockers (ARBs), statins, diuretics, calcium channel blockers, nitrates, insulin and other antidiabetic agents. Administering four evidence-based medications (EBMs; antiplatelet agents, beta-blockers, ACEIs/ARBs, statins) after AMI is considered a standard of care since 2004 and was included as a covariate in our analyses (yes/no). A variable was created summarizing the occurrence of in-hospital complications, such as cardiac arrest, pulmonary edema, bradycardia, re-infarction, ventricular tachycardia, ventricular fibrillation or cardiogenic shock (yes/no). A reduced left ventricular ejection fraction (LVEF) was noted if echocardiography, ventriculography or radionuclide ventriculography revealed a LVEF of < 30% (yes/no).

The outcome of this study was all-cause mortality after more than 28 days following AMI. It was determined by monitoring the vital status of study participants through population registries in- and outside the study region until 31 December 2011.

### Statistical analyses

Categorical variables were expressed as percentages, continuous variables as mean value with standard deviation (SD) and continuous variables that proved not to be normally distributed as median with interquartile range (IQR). Potential covariates were cross-tabulated with the five SPC groups. Differences in frequencies were tested using Chi^2^ or Kruskal-Wallis rank-sum test. To evaluate age differences among the SPC groups, a one-way ANOVA (analysis of variance) was performed. Kaplan-Meier plots were generated along with bivariate log-rank tests against survival to test for statistical significance.

To analyze the association between SPC and long-term mortality, Cox proportional hazards regression models were constructed. Patients with a SPC of 4.0 to <4.5 mEq/l served as reference group. An unadjusted model followed by a minimally adjusted model, additionally including the covariates sex and age, were calculated. A parsimonious model was created using backward elimination. Covariates that proved to be significantly associated with mortality in the univariate analysis were entered into the full model. Covariates were only included into the parsimonious model if they made a statistically significant contribution (*p* < 0.05) to the model. The final model was adjusted for prior angina pectoris, hypertension, hyperlipidemia, stroke, smoking status, peak CK-MB, any revascularization treatment and all four EBMs at discharge as well as the following discharge medications: diuretics, calcium channel blockers and insulin. The assumption of proportional hazards (parallel lines of log (−log(event)) versus log of event times) was tested graphically. Covariates violating the proportional hazards assumption were included as time-dependent covariates into the full model prior to backwards elimination. The covariates sex and age were forced to stay in the models. Multicollinearity among the independent variables was examined by assessing variance inflation factors (VIF) in the full model prior to backward selection [19].

Additionally, parsimonious models were calculated for observation periods of one, three, five and ten years in order to detect potential changes in HRs. A Cox regression model was calculated for patients excluded from the study due to missing data on covariates as a sensitivity analysis. To ensure comparability with the results of our study population, we adjusted this model for all covariates that were included in our main parsimonious model. Covariates providing information on stroke prior to AMI, smoking status and peak CK-MB were responsible for 95.3% (*n* = 875) of all missing values (*n* = 918) and, thus, could not be included in this model. Finally, since information on eGFR were only available from 2005 onwards, we calculated a separate Cox regression model using backward elimination and only including patients who were enrolled from 2005 onwards.

Statistical test results were considered significant if the *p* value was <0.05. All statistical analyses were performed using SAS software, version 9.2 (SAS Institute).

## Results

The study sample comprised 3347 patients with a first ever AMI and a mean age of 59.9 years (SD 9.8). Male patients accounted for 75.6% (*n* = 2531) of the sample. The median follow-up time was 6.1 years (IQR 4.2).

### Baseline characteristics

Baseline characteristics according to SPC are shown in Table 1. On average, patients with AMI had a SPC of 4.1 mEq/l (SD 0.5). The five SPC groups significantly differed from each other in terms of sex, history of diabetes and hypertension, smoking status, AMI type, laboratory values, medications received at hospital discharge (diuretics, insulin and other antidiabetic agents) and occurrence of any in-hospital complications (see Table 1).

**Table 1:** Baseline characteristics of patients with acute myocardial infarction by admission serum potassium concentration (*n* = 3347)

	Admission SPC, mEq/l					*p* Value
	<3.5 (*n* = 249)	3.5 - <4.0 (*n* = 1060)	4.0 - <4.5 (*n* = 1406)	4.5 - <5.0 (*n* = 498)	≥5.0 (*n* = 134)	
Sociodemographic characteristics						
Female, n (%)	99 (39.8)	276 (26.0)	306 (21.8)	103 (20.7)	32 (23.9)	<0.0001
Age (years), mean ± SD	60.1 ± 9.6	59.9 ± 9.9	59.6 ± 9.7	60.4 ± 9.8	61.1 ± 9.1	0.3156
Risk factors and co-morbidities, n (%)						
Angina pectoris	31 (12.5)	135 (12.7)	196 (13.9)	72 (14.5)	23 (17.2)	0.5858
Hypertension	199 (79.9)	813 (76.7)	1033 (73.5)	383 (76.9)	111 (82.8)	0.0278
Hyperlipidemia	173 (69.5)	737 (69.5)	998 (71.0)	336 (67.5)	99 (73.9)	0.5139
Stroke	12 (4.8)	56 (5.3)	77 (5.5)	29 (5.8)	14 (10.5)	0.1640
Diabetes mellitus^a^	59 (23.8)	273 (25.8)	376 (26.7)	172 (34.5)	58 (43.3)	<0.0001
Smoking status						
Current smoker	83 (33.3)	404 (38.1)	552 (39.3)	203 (40.8)	52 (38.8)	0.0140
Ex-smoker	71 (28.5)	318 (30.0)	423 (30.1)	156 (31.3)	55 (41.0)	
Never-smoker	95 (38.2)	338 (31.9)	431 (30.7)	139 (27.9)	55 (20.2)	
Clinical characteristics, n (%)						
AMI type^b^						
STEMI	125 (50.4)	489 (46.5)	555 (39.8)	187 (28.3)	47 (35.3)	0.0007
NSTEMI	114 (46.0)	516 (49.1)	768 (55.1)	267 (54.7)	79 (59.4)	
Bundle branch block	9 (3.6)	46 (4.4)	70 (5.0)	34 (7.0)	7 (5.3)	
LVEF <30%^c^	16 (9.5)	84 (10.9)	104 (10.5)	49 (13.7)	13 (14.6)	0.3453
Laboratory values, median (IQR)						
Admission creatinine (mg/dl)^d^	0.96 (0.8–1.2)	0.97 (0.8–1.1)	0.97 (0.8–1.1)	1.01 (0.8–1.2)	1.14 (1.0–1.5)	<0.0001
Admission hemoglobin (g/l)^d^	141 (131–151)	145 (136–155)	146 (137–156)	145 (135–154)	145 (129–158)	0.0079
Admission troponin-I (ng/ml)^e^	0.32 (0.1–2.7)	0.41 (0.1–3.2)	0.66 (0.1–3.8)	0.73 (0.2–5.9)	1.21 (0.2–4.9)	<0.0001
Peak CK-MB (U/l)	51 (15–122)	48 (18–119)	41 (16–105)	38 (15–99)	41 (15–84)	0.0462
Admission glucose (mg/dl)	143 (119–168)	130 (112–162)	126 (108–161)	131 (112–179)	152 (121–224)	<0.0001
eGFR (ml/min/1.73m^2^)^d^	76.2 (61.2–96.8)	78.9 (66.2–94.0)	80.4 (67.3–95.1)	77.3 (61.1–91.7)	65.8 (47.2–80.3)	<0.0001
eGFR <60 (ml/min/1.73m^2^), n (%)^d^	29 (23.6)	69 (14.2)	96 (15.3)	56 (23.5)	24 (40.0)	<0.0001
In-hospital treatment, n (%)						
Coronary angiography	237 (95.2)	1002 (94.5)	1331 (94.7)	458 (92.0)	120 (89.6)	0.0306
PCI^f^	170 (68.3)	757 (71.4)	970 (69.0)	339 (68.1)	84 (62.7)	0.2392
CABG	37 (14.9)	153 (14.4)	247 (17.6)	87 (17.5)	22 (16.4)	0.2638
Thrombolysis^g^	38 (18.9)	137 (18.0)	130 (14.0)	46 (13.7)	8 (9.2)	0.0338
Any revascularization treatment	212 (85.1)	934 (88.1)	1225 (87.1)	427 (85.7)	108 (80.6)	0.1199
Medication at hospital discharge						
Antiplatelet agents	236 (94.8)	1028 (97.0)	1357 (96.5)	484 (97.2)	128 (95.5)	0.4053
Beta-blockers	236 (94.8)	1023 (96.5)	1350 (96.0)	474 (95.2)	124 (92.5)	0.1863
ACEIs/ARBs	217 (87.2)	883 (83.3)	1151 (81.9)	425 (85.3)	108 (80.6)	0.1436
Statins	219 (88.0)	949 (89.5)	1263 (89.8)	443 (89.0)	115 (85.8)	0.6086
All four EBMs	176 (70.7)	763 (72.0)	1004 (71.4)	364 (73.1)	85 (63.4)	0.2770
Diuretics	147 (59.0)	533 (50.3)	643 (45.7)	247 (49.6)	86 (64.2)	<0.0001
Calcium channel blockers	41 (16.5)	120 (11.3)	174 (12.4)	51 (10.2)	22 (16.4)	0.0598
Nitrates	3 (1.2)	38 (3.6)	61 (4.3)	22 (4.4)	8 (6.0)	0.1066
Insulin	19 (7.6)	66 (6.2)	128 (9.1)	56 (11.2)	27 (20.2)	<0.0001
Other antidiabetic agents	11 (4.4)	103 (9.7)	149 (10.6)	84 (16.9)	23 (17.2)	<0.0001
Any In-hospital complications^h, i^, n (%)	53 (21.5)	162 (15.4)	201 (14.4)	89 (17.9)	24 (18.3)	0.0340

SPC, Serum potassium concentration; SD, Standard deviation; AMI, Acute myocardial infarction; STEMI, ST-elevation myocardial infarction; NSTEMI, non-ST-elevation myocardial infarction; LVEF, Left-ventricular ejection fraction; IQR, Interquartile range, CK-MB, Creatine kinase-myocardial band; eGFR, Estimated glomerular filtration rate; PCI, Percutaneous coronary intervention; CABG, Coronary artery bypass graft; ACEIs, Angiotensin-converting enzyme inhibitors; ARBs, Angiotensin-receptor blockers; EBMs, Evidence-based medications (antiplatelet agents, beta-blockers, ACEIs/ARBs, statins)

^a^ *n* = 3346

^b^ *n* = 3313

^c^ *n* = 2382

^d^ *n* = 1534

^e^ *n* = 2503

^f^ *n* = 3346

^g^ *n* = 2317

^h^ Including cardiac arrest, pulmonary edema, bradycardia, re-infarction, ventricular tachycardia, ventricular fibrillation and cardiogenic shock occurring during hospital stay

^i^ *n* = 3317

### Serum potassium concentration and long-term mortality

**Fig. 1:**
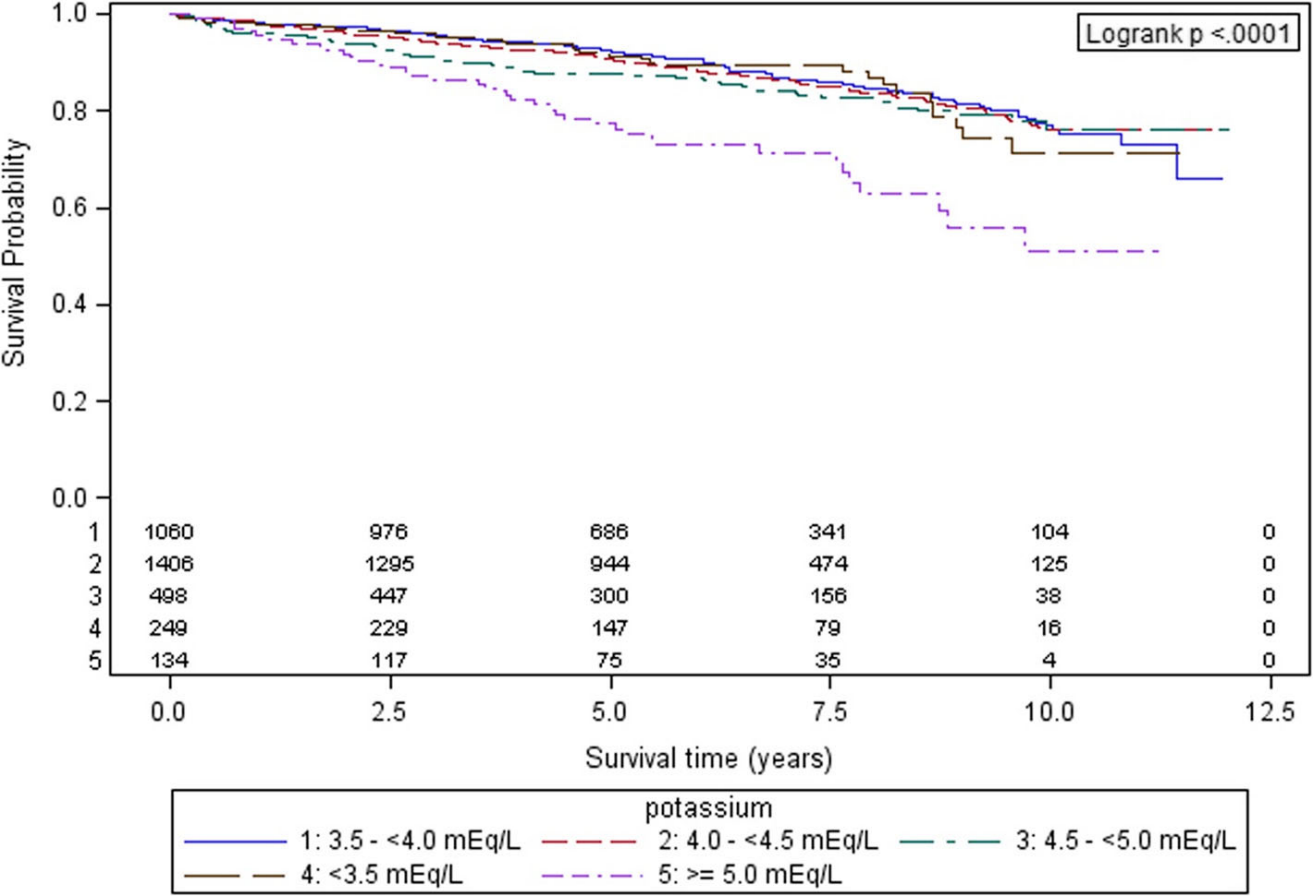
Kaplan-Meier curves of 12-year survival for the five admission serum potassium concentration groups

Long-term mortality in the whole study population was 14.3% (*n* = 481). The highest mortality of 29.9% (*n* = 40) was observed in patients with SPC of ≥5.0 mEq/l and the lowest (12.6%, *n* = 134) in patients with SPC of 3.5 to <4.0 mEq/l. Kaplan-Meyer survival curves along with the corresponding log-rank test demonstrated statistically significant differences in survival between the five serum potassium groups (see Fig. 1).

Results of the Cox regression analyses are shown in Table 2. In the unadjusted model, patients with SPC of ≥5.0 mEq/l had a significantly increased mortality risk compared to the reference group (unadjusted HR 2.49, 95% CI 1.77–3.50). Adjusting for sex and age resulted in a slight decrease of the HRs. Further adjusting for hypertension, hyperlipidemia, stroke, smoking status, peak CK-MB, any in-hospital revascularization, all four EBMs at discharge as well as diuretics, calcium channel blockers and insulin treatment at discharge, patients with SPC of ≥5.0 mEq/l still were at an increased risk of dying (adjusted HR 1.46, 95% CI 1.03 to 2.07). Patients with SPC of 3.5 to <4.5 mEq/l and <3.5 mEq/l showed the lowest long-term mortality risk compared to the reference group. However, these results did not prove to be statistically significant.

**Table 2:** Cox regression models for long-term mortality following acute myocardial infarction by admission serum potassium concentration (*n* = 3347)

Admission SPC, mEq/l	Unadjusted Model		Minimal Model^a^		Parsimonious Model^b^	
	HR (95% CI)	*p* Value	HR (95% CI)	*p* Value	HR (95% CI)	*p* Value
<3.5	0.98 (0.67–1.42)	0.9125	0.97 (0.68–1.44)	0.9420	0.92 (0.63–1.34)	0.6686
3.5 - <4.0	0.93 (0.74–1.15)	0.4871	0.88 (0.71–1.10)	0.2593	0.87 (0.70–1.09)	0.2297
4.0 - <4.5	1 (Ref.)		1 (Ref.)		1 (Ref.)	
4.5 - <5.0	1.18 (0.91–1.53)	0.2176	1.15 (0.88–1.49)	0.3038	1.08 (0.83–1.41)	0.5582
≥5.0	2.49 (1.77–3.50)	<0.0001	2.31 (1.64–3.25)	<0.0001	1.46 (1.03–2.07)	0.0360

SPC, Serum potassium concentration; HR, Hazard Ratio; CI, Confidence Interval

^a^Adjusted for sex and age

^b^Adjusted for sex, age, angina pectoris, hypertension, hyperlipidemia, stroke, smoking status, peak creatine kinase - mycoardial band (CK-MB), any revascularization treatment (coronary artery bypass surgery, percutaneous coronary intervention (PCI) or thrombolysis), all four evidence-based medications (EBMs) at discharge (antiplatelet agents, beta-blockers, ACEIs/ARBs (Angiotensin-converting enzyme inhibitors/Angiotensin receptor blockers), statins), diuretics at discharge, calcium channel blockers at discharge, insulin at discharge

In the analysis of patients who were enrolled from 2005 onwards, those with a SPC of 4.5 - <5.0 mEq/l and those with a SPC ≥5.0 mEq/l did not have significantly increased HRs. Additionally, patients with a SPC of 3.5 - <4.0 mEq/l had a significantly decreased mortality risk compared to the reference group (data not shown). Except for the hypertension and angina pectoris, the same covariates remained in the model as in the parsimonious model shown in Table 2.

#### 1-, 3-, 5- and 10-year mortality risks

Table 3 shows the results of the parsimonious regression models calculated for different observation periods as well as the corresponding mortality for each SPC group. The highest mortality was found in patients with a SPC of ≥5.0 mEq/l across all observation periods, whereas the lowest mortality was mainly found in patients with a SPC between 3.5 and <4.0 mEq/l. After a one-year observation period, the mortality risk of patients with a SPC between 4.5 and <5.0 mEq/l was increased by 96% (adjusted HR 1.96, 95% CI 1.10 to 3.48). Although the results for three- and five-year observation periods did not meet statistical significance, a trend towards an increased mortality risk in patients with SPC between 4.5 and <5.0 mEq/l could be identified (Table 3). After a ten-year observation period, only the HRs of patients with a SPC of ≥5.0 mEq/l were significantly increased.

**Table 3:** Adjusted Cox regression models for observation periods of one, three, five and ten years (*n* = 3347)

	Admission SPC, mEq/l	Parsimonious model^a^	
		HR (95% CI)	*p* Value
1-year observation period (>28 days to one year)	< 3.5	1.04 (0.43–2.55)	0.9269
	3.5 - <4–0	0.92 (0.51–1.67)	0.7924
	4.0 - <4.5	1 (Ref.)	
	4.5 - <5.0	1.96 (1.10–3.48)	0.0230
	≥5.0	1.22 (0.49–3.04)	0.6638
3-year observation period (>28 days to 3 years)	<3.5	0.60 (0.31–1.16)	0.1268
	3.5 - <4–0	0.72 (0.50–1.04)	0.0780
	4.0 - <4.5	1 (Ref.)	
	4.5 - <5.0	1.39 (0.96–2.00)	0.0799
	≥5.0	1.29 (0.75–2.21)	0.3560
5-year observation period (>28 days to 5 years)	<3.5	0.79 (0.49–1.29)	0.3423
	3.5 - <4–0	0.78 (0.58–1.04)	0.0901
	4.0 - <4.5	1 (Ref.)	
	4.5 - <5.0	1.32 (0.97–1.80)	0.0811
	≥5.0	1.40 (0.91–2.14)	0.1232
10-year observation period (>28 days to 10 years)	<3.5	0.92 (0.63–1.34)	0.6527
	3.5 - <4–0	0.85 (0.68–1.06)	0.1463
	4.0 - <4.5	1 (Ref.)	
	4.5 - <5.0	1.08 (0.83–1.41)	0.5650
	≥5.0	1.44 (1.02–2.05)	0.0218

SPC, Serum potassium concentration; HR, Hazard ratio; CI, Confidence interval

^a^Adjusted for sex, age, angina pectoris, hypertension, hyperlipidemia, stroke, smoking status, peak creatine kinase -myocardial band (CK-MB), any revascularization treatment (coronary artery bypass surgery, percutaneous coronary intervention (PCI) or thrombolysis), all four medications at discharge (antiplatelet agents, beta-blockers, ACEIs/ARBs (Angiotensin-converting enzyme inhibitors/Angiotensin receptor blockers), statins), diuretics at discharge, calcium channel blockers at discharge, insulin at discharge

### Mortality in patients excluded from the study

In patients excluded from the study due to partly missing data on relevant covariates, an increased long-term mortality risk was found in patients with SPC between 4.5 and <5.0 mEq/l (adjusted HR 1.43, 95% CI 1.00 to 2.04) and ≥5.0 mEq/l (adjusted HR 2.40, 95% CI 1.55 to 3.67) compared to the reference group (see Table 4).

**Table 4:** Adjusted Cox regression model for patients excluded from the study due to missing covariates (*n* = 875)

Admission SPC, mEq/l	Parsimonious Model ^a,b^	
	HR (95% CI)	*p* Value
<3.5	1.37 (0.80–2.33)	0.2481
3.5 - <4–0	1.03 (0.73–1.44)	0.8840
4.0 - <4.5	1 (Ref.)	
4.5 - <5.0	1.43 (1.00–2.04)	0.0486
≥5.0	2.40 (1.55–3.67)	<0.0001

SPC, Serum potassium concentration; HR, Hazard ratio; CI, Confidence interval

^a^Adjusted for age, sex, angina pectoris, hypertension, hyperlipidemia, any revascularization therapy (coronary artery bypass surgery, percutaneous coronary intervention (PCI) or thrombolysis), all four evidence-based medications (EBMs) at discharge (antiplatelet agents, beta-blockers, ACEIs/ARBs (Angiotensin-converting enzyme inhibitors/Angiotensin receptor blockers), statins), diuretics at discharge, calcium channel blockers at discharge, insulin at discharge

^b^ Not adjusted for stroke, smoking status and peak creatine kinase - myocardial band (CK-MB)

## Discussion

In the present study, we analyzed the association between admission SPC and long-term all-cause mortality in patients with AMI. Mortality risks were significantly increased in the highest SPC group (≥5.0 mEq/l). Analyses covering different observation periods showed a trend towards increased mortality risks in patients with SPC between 4.5 and <5.0 mEq/l as well as a significant association between a SPC of ≥5.0 mEq/l and increased mortality after a ten-year observation period.

In contrast to recent observational studies [8–10], our results for the total observation period do not suggest changing current clinical practice guidelines regarding desirable SPC in patients with AMI. A significantly increased long-term mortality risk was found only in patients with SPC of ≥5.0 mEq/l. However, our findings for shorter observation periods are partly comparable to the ones reported in recent studies. A study in 38,689 patients with AMI recruited from the Cerner Health Facts database and a Korean study in 1924 patients with AMI reported U-shaped associations between mean SPC and in-hospital and three-year mortality and found significantly increased risks in both patients with mean SPC of <3.5 mEq/l and ≥4.5 mEq/l [8, 9]. In addition to mean SPC, Goyal et al. analyzed the association between SPC measured at hospital admission and in-hospital mortality [8]. The HRs were attenuated yet still significantly increased in patients with SPC ≥4.5 mEq/l. In line with those findings, patients with SPC between 4.5 and <5.0 mEq/l had an increased mortality risk in our study population after a one-year observation period. A similar trend, yet not statistically significant, was found for three- and five-year observation periods. Our analysis of patients excluded from the study also suggests that an increased risk of dying might already be present in patients with SPC between 4.5 and <5.0 mEq/l. In patients with AMI, SPC between 4.5 and <5.0 mEq/l might therefore negatively affect survival during the first few years following the event, whereas in the long run, SPC of ≥5.0 mEq/l might be more harmful. A similar trend was demonstrated in another study in AMI patients [10]. In this study, the odds ratios of patients with SPC between 4.5 and ≤5.0 mEq/l were significantly increased after one and five years of follow-up. However, the OR deviated only marginally from the reference group after a ten-year follow-up [10].

Studies conducted in prior years examined in-hospital outcomes associated with SPC, but had low statistical power to detect higher mortality risks due to small study populations. These studies concluded that patients with AMI and hypokalemia had an increased risk for cardiac arrhythmias [20, 21] and a higher in-hospital mortality [22]. One study found no significant difference in in-hospital mortality in patients with AMI and hypo- or normokalemia [23]. Comparability with these studies is limited not only because they focused on in-hospital outcomes, but also due to methodological differences and the fact that AMI treatment has changed substantially both in terms of revascularization and drug treatment.

In contrast to earlier studies [7–9, 22, 24], we did not detect increased mortality risks in patients with low SPC. These results might be explained by an improved treatment of patients with AMI counteracting hypokalemia. Apart from beneficial effects on survival, medications routinely administered after AMI, such as beta-blockers, prevent hypokalemia [8, 23]. Furthermore, hypokalemia affects morbidity and mortality in patients with an established cardiovascular event [7, 22, 25, 26]. Especially patients with AMI had an increased risk for ventricular arrhythmias even if their SPC was only mildly decreased [1, 3, 7]. Additionally, changes in SPC subsequent to hospital admission occur frequently. A recent study in patients with heart failure concluded that the SPC measured within 48 h after hospital admission is often abnormal and increases during hospitalization [27]. SPC in our study was only measured at hospital admission and we did not know if and how hypo-or hyperkalemia were treated during hospital stay. Improved medical treatment of AMI and of hypokalemia in AMI patients might have positively influenced long-term survival in our study population with low SPC at hospital admission.

Potassium homeostasis primarily depends on a normal renal function [7] and both hypo- and hyperkalemia can occur due to impaired renal excretion [1, 2]. According to a large-scale study in 118,753 patients with AMI, renal impairment is an important long-term predictor of mortality [28]. Furthermore, hyperkalemia has been demonstrated to be one of the largest risk factors for all-cause mortality in patients with an established cardiovascular disease and impaired renal function [29]. In our study population, patients with high SPC had a lower eGFR than the reference group, indicating renal impairment. However, the corresponding covariate did not make it into the final parsimonious model. Nevertheless, we cannot rule out the possibility of bias since the admission creatinine level, and consequently the eGFR, was only available from 2005 onwards in our data set. In the analysis including only patients enrolled from 2005 onwards, we did not find significantly increased mortality risks in those with a SPC of 4.5- <5.0 mEq/l and ≥5.0 mEq/l, respectively.

In the present study we focused on long-term mortality and, therefore, patients who died within 28 days after AMI were excluded. The excluded patients had a SPC of 4.4 mEq/l (SD 0.8), while the actual study population had a mean SPC of 4.1 mEq/l (SD 0.5) (data not shown). Patients who died within 28 days were more likely to have extreme SPC (<3.5 mEq/l and ≥5.0 mEq/l), they were overall sicker and older than the included patients (data not shown). Therefore, including them into the analysis would have further increased the mortality risk estimates for patients with high SPC.

This study is characterized by several strengths. To our knowledge this is the first longitudinal study with an observation period of more than three years. Data were collected in the framework of a population-based registry with consecutive enrollment. Important covariates such as in-hospital treatment and complications, medication received at hospital discharge as well as risk factors and co-morbidities were included.

The following limitations should be considered. We did not have the possibility to distinguish between potassium-sparing versus non-potassium-sparing diuretics. In addition, our results do not apply to patients with AMI who are older than 74 years. Data on ethnicity was not collected in the framework of the registry and, therefore, the results might not be generalizable to all ethnic groups. Patients with AMI were enrolled between 2000 and 2008 for this study and treatment strategies most likely improved during this time span both for hypo- or hyperkalemia after AMI as well as AMI itself. Data on the treatment of AMI was included in the analysis; however, data on the treatment of abnormal SPC was not collected in the framework of the registry. Finally, due to the observational design of our study we might not have considered all relevant confounders and we cannot exclude the possibility of reverse causation.

## Conclusion

Based on our analysis, an admission SPC of ≥5.0 mEq/l might be associated with an increased mortality risk in patients with AMI. Furthermore, our results indicate that also patients with admission SPC between 4.5 and <5.0 mEq/l may experience a higher mortality risk in the first few years following AMI. Due to the limited data on renal function in our study, further long-term studies are needed to provide evidence on the relevance of admission SPC in patients with AMI.

## Abbreviations

ACEI: Angiotensin-converting enzyme inhibitor
AMI: Acute myocardial infarction
ANOVA: Analysis of variance
ARB: Angiotensin-receptor blockers
CK-MB: Creatine kinase-myocardial band
EBM: Evidence-based medication
eGFR: Estimated glomerular filtration rate
HR: Hazard ratio
IQR: Interquartile range
KORA: Cooperative Health Research in the Region of Augsburg
LVEF: Left ventricular ejection fraction
MDRD: Modification of Diet in Renal Disease
MI: Myocardial infarction
MONICA: Monitoring Trends and Determinants in Cardiovascular disease
PCI: Percutaneous coronary intervention
SD: Standard deviation
SPC: Serum potassium concentration
VIF: Variance inflation factor
WHO: World Health Organization

## References

[1] Schaefer TJ, Wolford RW (2005). Disorders of potassium. Emerg Med Clin North Am.

[2] Clausen T (2010). Hormonal and pharmacological modification of plasma potassium homeostasis. Fundam Clin Pharmacol.

[3] Alfonzo AV, Isles C, Geddes C, Deighan C (2006). Potassium disorders--clinical spectrum and emergency management. Resuscitation.

[4] Cohn JN, Kowey PR, Whelton PK, Prisant LM (2000). New guidelines for potassium replacement in clinical practice: a contemporary review by the National Council on potassium in clinical practice. Arch Intern Med.

[5] Zipes DP, Camm AJ, Borggrefe M, Buxton AE, Chaitman B, Fromer M, Gregoratos G, Klein G, Moss AJ, Myerburg RJ *et al*. ACC/AHA/ESC 2006 guidelines for management of patients with ventricular arrhythmias and the prevention of sudden cardiac death: a report of the American College of Cardiology/American Heart Association Task Force and the European Society of Cardiology Committee for Practice Guidelines (Writing Committee to Develop guidelines for management of patients with ventricular arrhythmias and the prevention of sudden cardiac death) developed in collaboration with the European Heart Rhythm Association and the Heart Rhythm Society. Europace. 2006;8(9):746–837. 10.1093/europace/eul108 16935866

[6] Antman EM, Anbe DT, Armstrong PW, Bates ER, Green LA, Hand M, Hochman JS, Krumholz HM, Kushner FG, Lamas GA (2004). ACC/AHA guidelines for the management of patients with ST-elevation myocardial infarction--executive summary. A report of the American College of Cardiology/American Heart Association task force on practice guidelines (writing committee to revise the 1999 guidelines for the management of patients with acute myocardial infarction). J Am Coll Cardiol.

[7] Macdonald JE, Struthers AD (2004). What is the optimal serum potassium level in cardiovascular patients?. J Am Coll Cardiol.

[8] Goyal A, Spertus JA, Gosch K, Venkitachalam L, Jones PG, Van den Berghe G, Kosiborod M (2012). Serum potassium levels and mortality in acute myocardial infarction. JAMA.

[9] Choi JS, Kim YA, Kim HY, Oak CY, Kang YU, Kim CS, Bae EH, Ma SK, Ahn YK, Jeong MH (2014). Relation of serum potassium level to long-term outcomes in patients with acute myocardial infarction. Am J Cardiol.

[10] Shiyovich A, Gilutz H, Plakht Y (2014). Serum potassium levels and long-term post-discharge mortality in acute myocardial infarction. Int J Cardiol.

[11] Meisinger C, Hormann A, Heier M, Kuch B, Lowel H (2006). Admission blood glucose and adverse outcomes in non-diabetic patients with myocardial infarction in the reperfusion era. Int J Cardiol.

[12] Kuch B, Heier M, von Scheidt W, Kling B, Hoermann A, Meisinger C (2008). 20-year trends in clinical characteristics, therapy and short-term prognosis in acute myocardial infarction according to presenting electrocardiogram: the MONICA/KORA AMI registry (1985-2004). J Intern Med.

[13] Kirchberger I, Heier M, Goluke H, Kuch B, von Scheidt W, Peters A, Meisinger C (2016). Mismatch of presenting symptoms at first and recurrent acute myocardial infarction. From the MONICA/KORA myocardial infarction registry. Eur J Prev Cardiol.

[14] Lowel H, Meisinger C, Heier M, Hormann A (2005). The population-based acute myocardial infarction (AMI) registry of the MONICA/KORA study region of Augsburg. Gesundheitswesen.

[15] Levey AS, Bosch JP, Lewis JB, Greene T, Rogers N, Roth D (1999). A more accurate method to estimate glomerular filtration rate from serum creatinine: a new prediction equation. Modification of diet in renal disease study group. Ann Intern Med.

[16] Matsushita K, Van Der Velde M, Astor BC, Woodward M, Levey AS, De Jong PE, Coresh J, Gansevoort RT, Chronic Kidney Disease Prognosis Consortium (2010). Association of estimated glomerular filtration rate and albuminuria with all-cause and cardiovascular mortality in general population cohorts: a collaborative meta-analysis. Lancet.

[17] Bae EH, Lim SY, Cho KH, Choi JS, Kim CS, Park JW, Ma SK, Jeong MH, Kim SW (2012). GFR and cardiovascular outcomes after acute myocardial infarction: results from the Korea acute myocardial infarction registry. Am J Kidney Dis.

[18] Chin CT, Wang TY, Li S, Wiviott SD, DeLemos JA, Kontos MC, Peterson ED, Roe MT. comparison of the prognostic value of peak creatine kinase-MB and troponin levels among patients with acute myocardial infarction: a report from the acute coronary treatment and intervention outcomes network registry-get with the guidelines. Clin Cardiol 2012;35(7):424–429. 10.1002/clc.21980 PMC6652484 22434769

[19] Allison P (2012). When can you safely ignore multicollinearity?.

[20] Friedensohn A, Faibel HE, Bairey O, Goldbourt U, Schlesinger Z (1991). Malignant arrhythmias in relation to values of serum potassium in patients with acute myocardial infarction. Int J Cardiol.

[21] Nordrehaug JE, Johannessen KA, von der Lippe G (1985). Serum potassium concentration as a risk factor of ventricular arrhythmias early in acute myocardial infarction. Circulation.

[22] Dyckner T (1990). Relation of cardiovascular disease to potassium and magnesium deficiencies. Am J Cardiol.

[23] Madias JE, Shah B, Chintalapally G, Chalavarya G, Madias NE (2000). Admission serum potassium in patients with acute myocardial infarction: its correlates and value as a determinant of in-hospital outcome. Chest.

[24] Krijthe BP, Heeringa J, Kors JA, Hofman A, Franco OH, Witteman JC, Stricker BH (2013). Serum potassium levels and the risk of atrial fibrillation: the Rotterdam study. Int J Cardiol.

[25] Hulting J (1981). In-hospital ventricular fibrillation and its relation to serum potassium. Acta Med Scand Suppl.

[26] Pourmoghaddas A, Shemirani H, Garakyaraghi M (2012). Association of serum potassium level with ventricular tachycardia after acute myocardial infarction. ARYA Atheroscler.

[27] Khan SS, Campia U, Chioncel O, Zannad F, Rossignol P, Maggioni AP, Swedberg K, Konstam MA, Senni M, Nodari S (2015). Changes in serum potassium levels during hospitalization in patients with worsening heart failure and reduced ejection fraction (from the EVEREST trial). Am J Cardiol.

[28] Smith GL, Masoudi FA, Shlipak MG, Krumholz HM, Parikh CR (2008). Renal impairment predicts long-term mortality risk after acute myocardial infarction. J Am Soc Nephrol.

[29] Jain N, Kotla S, Little BB, Weideman RA, Brilakis ES, Reilly RF, Banerjee S (2012). Predictors of hyperkalemia and death in patients with cardiac and renal disease. Am J Cardiol.

